# If Not COVID-19 What Is It? Analysis of COVID-19 versus Common Respiratory Viruses among Symptomatic Health Care Workers in a Tertiary Infectious Disease Referral Hospital in Manila, Philippines

**DOI:** 10.3390/tropicalmed6010039

**Published:** 2021-03-19

**Authors:** Kristal An Agrupis, Annavi Marie G. Villanueva, Ana Ria Sayo, Jezreel Lazaro, Su Myat Han, Alyannah C. Celis, Shuichi Suzuki, Ann Celestyn Uichanco, Jocelyn Sagurit, Rontgene Solante, Lay-Myint Yoshida, Koya Ariyoshi, Chris Smith

**Affiliations:** 1 San Lazaro Hospital, Nagasaki University Collaborative Research Office, Manila 1003, Philippines; agrupiskristalan@gmail.com (K.A.A.); anyacloud23@gmail.com (A.C.C.); suzuki_shuichi@nagasaki-u.ac.jp (S.S.); 2 School of Tropical Medicine and Global Health, Nagasaki University, Nagasaki 852-8102, Japan; pearl.june@gmail.com (S.M.H.); koya.ariyoshi@gmail.com (K.A.); christopher.smith@lshtm.ac.uk (C.S.); 3 Adult Infectious Disease and Tropical Medicine Department, San Lazaro Hospital, Manila 1003, Philippines; anariasayo@yahoo.com (A.R.S.); jez_lazaro@yahoo.com (J.L.); acpuichanco@gmail.com (A.C.U.); berewmd@yahoo.com.ph (J.S.); rontgenesolante@gmail.com (R.S.); 4 Institute of Tropical Medicine, Nagasaki University, Nagasaki 852-8523, Japan; lmyoshi@nagasaki-u.ac.jp; 5 Faculty of Infectious and Tropical Diseases, London School of Hygiene and Tropical Medicine, London WC1E 7HT, UK

**Keywords:** COVID-19, respiratory viruses, coinfections, health care workers, Philippines, rhinovirus

## Abstract

The COVID-19 global pandemic is entering its second year. In this short report we present additional results as a supplement to our previous paper on COVID-19 and common respiratory virus screening for healthcare workers (HCWs) in a tertiary infectious disease referral hospital in Manila, Philippines. We sought to understand what etiologic agents could explain the upper/lower respiratory tract infection-like (URTI/LRTI-like) symptoms exhibited by 88% of the 324 HCWs tested. Among the patients who had URTI/LRTI-like symptoms, only seven (2%) were positive for COVID-19, while 38 (13%) of the symptomatic participants were identified positive for another viral etiologic agent. Rhinovirus was the most common infection, with 21 (9%) of the symptomatic participants positive for rhinovirus. Based on these results, testing symptomatic HCWs for common respiratory illnesses in addition to COVID-19 should be considered during this time of global pandemic.

## 1. Introduction

The COVID-19 global pandemic is caused by the novel severe acute respiratory syndrome coronavirus 2 (SARS-CoV-2) and continues into a second year [1]. In the Philippines, around ~552,000 cases have been recorded, ~11,500 (2%) of whom have died, as of 15 February 2021 [2]. HCWs remain at high risk of acquiring the infection as they attend hospitalized COVID-19 patients. In the months that followed the first confirmed case of COVID-19 in the Philippines, recorded in San Lazaro Hospital (SLH) [3], there has been an increasing national trend of confirmed cases. The influx of patients has placed an enormous workload on hospitals, and consequently to HCWs. Mandatory quarantine/isolation of HCWs suspected of exposure has strained hospital workforces; specifically, HCWs experiencing a high-risk exposure to a confirmed case, or presenting with influenza-like-illness (ILI), are required to undergo at least two weeks of quarantine/isolation away from the hospital. The resulting reduction in the HCW workforce causes extended work hours for the remaining available staff, and inevitable “shift fatigue” [4] accompanied with increased fear and anxiety in working with COVID-19 patients.

We have reported that at SLH in Manila only 2% of HCWs were positive for SARs-CoV-2 as screened by RT-PCR (8 out of 324 HCWs screened) [5]. At that time, the criteria for screening were: (1) a history of close contact or high-risk exposure with a confirmed COVID-19 case or (2) the development of COVID-related signs and symptoms. Of the 324 HCWs, 88% had upper/lower respiratory tract infection-like (URTI/LRTI-like) illness such as fever, cough, sore throat, runny nose, shortness of breath, and loss of smell and taste. The small percentage of SARs-CoV-2 positivity suggests the possibility of underlying infections other than SARs-CoV-2.

SARS-CoV-2 is projected to circulate in the population indefinitely [6], and thus is likely to continue to cocirculate with common respiratory viruses. Although there is limited data on the prevalence of common respiratory viral infections in the Philippines, the Department of Health reports that acute respiratory infections consistently top the leading causes of morbidity in the country [7]. COVID-19 resides in the respiratory tract of the human host, and commonly exhibits URTI/LRTI-like symptoms, which overlap with those of common respiratory viral infections [8]. Because of the relatively small proportion of confirmed COVID-19 among HCWs described above [5], we sought to investigate whether HCWs who presented with these symptoms could be explained by other common respiratory viral etiologic agents.

## 2. Materials and Methods

HCW participants were tested for the presence of the following respiratory viruses: COVID-19, influenza A and B, human metapneumovirus (HMPV), respiratory syncytial virus (RSV), parainfluenza types 1 to 4, coronavirus 229E, coronavirus OC43, rhinovirus, adenovirus, and bocavirus.

Viral RNA from nasopharyngeal and oropharyngeal swab specimens were extracted using a QIAamp Viral RNA Mini Kit (Qiagen, Hilden, Germany), following the manufacturer’s instructions [9]. Real-time PCR was performed to detect SARS-CoV-2 viral RNA using Corman et al. [10] and Nao et al. [11] protocols. Multiplex and hemi-nested multiplex PCR was done to test for the presence of the genetic materials from 13 other respiratory viruses, based on the protocol by Yoshida et al. [12]. Amplicons were visualized in 2% agarose gel.

We limited our analysis to those who exhibited URTI/LRTI-like symptoms. We categorized participants as “having URTI/LRTI-like symptoms” when they exhibited at least one of the following: fever, cough, sore throat, runny nose, shortness of breath, or loss of smell and taste. The epidemiological and clinical characteristics of these HCWs were compared between those who tested positive for the respiratory viral panels other than COVID-19 and those who tested negative for any of the panel of tests. The values were expressed as absolute numbers and percentages for categorical variables, and mean with standard deviation (SD) and median for continuous variables. Fisher’s exact test and chi-square test were used to test for associations between categorical variables, and the Mann−Whitney test was used to compare discrete variables between classifications of categorical variables. A p-value of ≤0.05 was considered statistically significant. Stata SE ver. 16.1 (StataCorp 2019, College Station, TX, USA) was used for all analyses.

## 3. Results

Our previous publication detailed the epidemiological and clinical characteristics of the 324 HCW staff tested for SARS-CoV-2 at SLH in Manila [5]. Here we subjected the same group to a panel of respiratory viral tests from March 20 to 20 April 2020. Out of 324, 286 (88%) presented with URTI/LRTI-like symptoms. A summary of those who tested positive for at least one of the viral panels, including SARS-CoV-2, is shown in Table 1. Among those who exhibited URTI/LRTI-like symptoms, only 2% (7 out of 286) were confirmed to have SARS-COV-2 infection. Specifically, five tested positive for only SARS-CoV-2 (2%) and two others had coinfections with two or more viruses: one with influenza A (0.4%), and another with influenza A and parainfluenza 1 (0.4%). Twenty-one (9%) of the participants were infected with rhinovirus—19 with rhinovirus alone (7%) and two with bocavirus coinfection (0.7%)—making it the most common infection of those who exhibited URTI/LRTI-like symptoms (55% out of those who yielded positive results). Other viruses that tested positive were influenza A (0.7%) and B (0.7%), bocavirus (0.4%), parainfluenza 1 (0.4%), adenovirus (0.7%), and coinfections of adenovirus and bocavirus (0.7%). Coinfection was observed in six HCW samples (2%). Overall, 38 (13%) of the participants who presented with URTI/LRTI-like symptoms were identified with a viral etiologic agent. HMPV, RSV, Parainfluenza 2 to 4, coronavirus 229E, and coronavirus OC43 were not detected in any of the samples.

Excluding SARS-CoV-2 from the analysis (Table 2), most participants who tested positive for the respiratory viral panel (N = 36) belonged to a young age group (39% were 20–29 years old, and 33% were 30–39 years old), were female (67%), and worked as nurses (67%). Other health care workers infected were six nursing aides (17%), four radiology technicians (11%), and two medical doctors (6%). The most common respiratory symptoms exhibited by those who tested positive were sore throat (71%), cough (50%), and runny nose (43%); while nonrespiratory symptoms were predominantly headache (58%), myalgia (33%), and fatigue (19%). Only headache showed significant association with being positive for the respiratory viruses tested (*p* = 0.04).

## 4. Discussion

In the present study more health care workers exhibiting URTI/LRTI-like symptoms tested positive for common respiratory viruses, particularly rhinoviruses [13], than pandemic-related COVID-19. Our results are comparable to the outcome of an earlier study from China which tested clinically suspected COVID-19 patients for SARS-CoV-2 and other respiratory viruses. Their findings showed that 1% were positive for SARS-CoV-2, with an overall detection rate of other respiratory pathogens of 10%, and rhinovirus also being the most common underlying virus (3%). However, the coinfection rate was higher at 12% [14]. Notably, coinfections in our samples were observed more commonly with bocavirus (four samples that had coinfections with other respiratory viruses). Two (<1%) confirmed SARS-CoV-2 positive samples also had coinfections. Reported rates of coinfection of SARS-CoV-2 and other respiratory viruses range from 1% to 20% [15,16,17].

Among those who had coinfections, only one HCW yielded positive results for three etiologic agents (SARS-CoV-2, Influenza A and Parainfluenza (1); a 48-year-old, male who was tested on the fifth day of illness. He was categorized as low-risk exposure and presented with fever, sore throat, cough, fatigue and loss of appetite.

The signs and symptoms exhibited by those who tested positive for respiratory viruses other than COVID-19 were indistinguishable from those observed among persons who tested positive for SARS-CoV-2 [5]. Based upon symptoms alone it is a challenge to identify patients with acute respiratory illness (ARI) and COVID-19, primarily because their presenting signs and symptoms are almost similar at the outset. In the context of HCWs who are attending to COVID-19 patients in a developing country like the Philippines, where hospitals are usually understaffed, it is crucial to clarify the actual infection rate of SARS-CoV-2 and other respiratory viruses. At the moment, for HCWs presenting with mild symptoms who tested negative for SARS-CoV-2, for example, this guides decisions regarding continuing quarantine versus allowing to work. In addition to relieving the burden of “shift fatigue” among HCWs, a definitive diagnosis of “something other than COVID-19” should also help alleviate unnecessary anxiety. A limitation of this study is that we tested for an incomplete panel of viruses, and no bacterial pathogens. Subclinical viral infection is common, particularly rhinovirus, among healthy individuals [18], and it is possible that the symptoms of the HCWs in our study could be explained by other causative agents than those in the respiratory viral panel we tested. Further studies could test for an expanded range of pathogens relevant to respiratory illness.

## 5. Conclusions

Although COVID-19 is a serious concern, our study showed that it was not the main pathogen responsible for respiratory tract infections among HCWs. Symptoms associated with SARS-CoV-2 and other respiratory viral etiologies are frequently shared. While it is important to continue regular screening and testing for COVID-19 among this high-risk population, efforts should also focus on ensuring that HCWs have access to rapid molecular tests for other common respiratory illnesses.

## Figures and Tables

**Table 1:** Summary of the positive test results of viral panels (including SARS-CoV-2) tested among HCWs suspected of having COVID-19 and presented with URTI/LRTI-like symptoms, San Lazaro Hospital, March−April 2020 (N = 286).

Viral Etiologic Agents	Infected HCWs (n, %)
SARS-CoV-2	5 (1.7)
Rhinovirus	19 (6.5)
Influenza A	2 (0.7)
Influenza B	2 (0.7)
Human bocavirus	1 (0.4)
Parainfluenza 1	1 (0.4)
Adenovirus	2 (0.7)
Coinfections *	
SARS-CoV-2+ Influenza A + Parainfluenza 1	1 (0.4)
SARS-CoV-2 + influenza A	1 (0.4)
Adenovirus + Bocavirus	2 (0.7)
Rhinovirus + Bocavirus	2 (0.7)
**Total**	**38 (13.3)**

* Coinfection—sample tested positive for more than one etiologic agent.

**Table 2:** Comparison of characteristics and signs and symptoms of HCWs suspected to have COVID-19 who tested positive and negative for the additional respiratory viral panel *.

Characteristics	Positive (N = 36) (n, %)	Negative (N = 280) (n, %)	*p*-Value
**Age (years)**			
mean (SD)	35 (11.1)	36 (8.4)	
median (range)	30.5 (23–63)	33 (24–61)	0.14
**Age group (years)**			
20–29	14 (38.9)	72 (25.7)	
30–39	12 (33.3)	124 (44.3)	
40–49	4 (11.1)	62 (22.1)	
50–59	4 (11.1)	20 (7.1)	
60–69	2 (5.6)	2 (0.7)	
**Sex**			
Female	24 (66.7)	186 (66.4)	
Male	12 (33.3)	94 (33.6)	1
**Occupation**			
Nurse	24 (66.7)	175 (62.5)	0.03
Medical doctor	2 (5.6)	34 (12.1)	
Nursing aide	6 (16.7)	6 (16.7)	
Radiology technician	4 (11.1)	2 (0.7)	
Laboratory personnel	0	9 (3.2)	
Admission/reception staff	0	4 (1.4)	
**Level of exposure**			
Low risk	28 (77.8)	195 (69.6)	0.3
High risk	8 (22.2)	85 (30.4)	
**URTI/LRTI-like symptoms**			
Fever	1 (2.8)	2 (0.7)	0.3
Cough	18 (50)	138 (50.4)	1
Sore throat	25 (71.4)	188 (68.6)	0.7
Runny nose	15 (42.9)	108 (39.4)	0.7
Shortness of breath	2 (5.7)	26 (9.6)	0.4
Loss of smell	3 (11.5)	7 (3.2)	0.1
Loss of taste	1 (3.9)	8 (3.7)	1
**Comorbidities**			
Asthma	2 (5.6)	20 (7.1)	1
Cancer	0	2 (0.7)	1
Chronic liver disease	0	1	0.4
Diabetes	1 (12.5)	5 (13.9)	0.92
Heart disease	2 (5.6)	6 (2.1)	0.2
Hypertension	8 (22.2)	58 (20.7)	0.8
Obesity	6 (16.7)	50 (17.9)	1
No comorbidities	20 (55.6)	172 (61.4)	0.5
**Duration between onset of symptoms and swab collection**			
mean (SD)	8 (5.4)	9 (8.1)	
median	7	6	0.5

* HCWs who tested positive for covid-19 were excluded from the analysis.
